# Protocol of the “*As du Coeur*” study: a randomized controlled trial on physical activity maintenance in cardiovascular patients

**DOI:** 10.1186/s12872-016-0325-0

**Published:** 2016-08-22

**Authors:** Marion Fournier, Rémi Radel, Karim Tifratene, Christian Pradier, Alain Fuch, Philippe Mossé, Jean-Jacques Domerego, Jocelyn Gal, Fabienne d’Arripe-Longueville

**Affiliations:** 1 Laboratoire LAMHESS EA 6312, University Nice Sophia Antipolis, Nice, France; 2 Department of Public Health, Centre Hospitalier Universitaire de Nice, Nice, France; 3 Régime Social des Indépendants, Nice, France; 4 Laboratoire LEST CNRS, University of Aix-Marseille, Nice, France; 5 Hôpital Privé Gériatrique les Sources, Nice, France; 6 Centre Antoine Lacassagne, Nice, France

**Keywords:** Habits, Physical activity, Cardiac rehabilitation, Randomized controlled trial

## Abstract

**Background:**

Although the benefits of supervised physical activity programs in cardiac rehabilitation have been well documented, the amount of physical activity often drops quickly after the end of the supervised period. This trial (registered as ISRCTN77313697) will evaluate the effectiveness of an experimental intervention based on habit formation theory applied to physical activity maintenance.

**Methods/Design:**

Cardiovascular patients (*N* = 56) will be individually randomized into two groups. Two supervised physical activity (SPA) sessions per week will be offered to the first group for 20 weeks. Progressively autonomous physical activity (PAPA) will be offered to the second group as follows: 10 weeks of the same supervised program as the SPA group followed by 10 more weeks in which one supervised session will be replaced by a strategy to build and sustain the habit of autonomous practice of physical activity. The primary outcome is the amount of physical activity measured by the International Physical Activity Questionnaire (IPAQ; Craig et al., Med Sci Sport Exercises 35(8):1381–95, 2003). To compensate for the limited capacity to recruit subjects, multiple IPAQ measurements will be made (at T0, T5, T7, T9 and T12 months after the start of the intervention) and analyzed using the mixed model approach. We will also assess changes in physical and physiological indicators, automaticity of the physical activity behavior, motivation and quality of life. Last, we will assess the cost-effectiveness for each type of program.

**Discussion:**

If proven to be effective, the PAPA intervention, which requires fewer supervised sessions, should provide a cost-effective solution to the problem of physical activity maintenance in cardiac rehabilitation.

## Background

Cardiovascular disease (CVD) is the leading cause of death worldwide, accounting for 30 % (16.7 million) of all deaths [45]. Coronary heart disease makes up the highest proportion of CVD mortalities and is projected to show an increase of 16.6 % by 2030 [24]. Strategies to reduce the impact of CVD have thus become crucial. Cardiac rehabilitation (CR) is a complex secondary prevention intervention to reduce of risk of cardiovascular disease and to promote the adoption and maintenance of healthful behaviors in CVD patients. Exercise has consistently been identified as an integral component of CR in the secondary prevention guidelines [3].

Supervised physical activity (PA) programs for CVD patients effectively improve the level of physical activity [42] but PA maintenance once supervision has ended is often disappointing, typically showing a linear decline over time. A comprehensive search of the literature on post-CR PA indicates that this behavior quickly drops when CR ends (e.g., [18]). In 2003, Yu et al. evaluated the long-term changes in exercise capacity in cardiac patients who participated in an 8-week center-based CR program. At the 2-year follow-up, the improved exercise capacity had declined to the point where it was similar to that of the inactive control group. A meta-analysis by Chase [12] reviewed 14 intervention programs to maintain or increase PA after CR. Although maintenance was enhanced using behavioral or cognitive methods, none of the programs focused on habit formation. Yet recent work has identified habit as the main predictor of maintaining health behaviors [20, 36]. While enacting a behavior that is regulated by motivation typically requires deliberate effort, habits are thought to be triggered automatically and may thus occur in the absence of awareness, conscious control, mental effort and deliberation [46]. This would explain why habits are particularly well-suited to guiding behavior in everyday life, as high cognitive demand (e.g., time pressure, distractions, stress, fatigue) reduces the capacity to act on deliberative processes.

Nevertheless, habit formation is a long process that requires many repetitions of the same behavior in the same context [26]. It is assumed that successful habit formation depends on the frequency and consistency of the contextual cues associated with the desired behavioral response [46]. A few studies have started to develop interventions based on the habit framework. In a recent trial [25], volunteers wanting to lose weight were randomized to one of two conditions: one in which a leaflet listing 10 simple diet and activity behaviors was provided, as well as encouragement for context-dependent repetition, and a control condition of a no-treatment waiting list. After 8 weeks, the intervention group had lost 2 kg compared with 0.4 kg in the control group. Interestingly, the effect did not vanish over time but became stronger, with a 3.8-kg loss for the intervention group at the end of the 32-week follow-up period. Moreover, actions that had been initially difficult to carry out became easier to maintain. Another randomized controlled trial recently replicated this effect on weight loss in a primary care setting with a larger sample over a 24-month follow-up period [5]. In the PA domain, a recent study (Phillips & Gardner, [33]) confirmed an earlier one (Verplanken & Melkevik [43]) demonstrating that an important factor of habitual exercise concerns the decision to go. Phillips and Gardner [33] confirmed this finding and called the decision exercise instigation. They further demonstrated that the habit strength of exercise instigation was the only predictor of exercise frequency.

Habit theory assumes that habits are context-dependent, and this might explain why behaviors learned in CR centers are hard to sustain outside the centers in the real living environment. These patients are often insufficiently physically active during leisure time prior to diagnosis (e.g., [16]). Moreover, upon diagnosis, they face psychological barriers to exercise related to fears of death, perceived exercise risks or lack of self-efficacy (e.g., [22]). While direct supervision in CR centers has the advantage of providing a safe environment with trained coaches and the possibility for social exchange, it does not reproduce the patients’ living context. At the end of center-based CR, patients return to their everyday environments, which more often than not are characterized by sedentary habits. Reviews comparing the level of PA in center-based versus home-based interventions have indeed shown that center-based interventions ultimately end with PA levels similar to those of home-based interventions once the program is over—and with lower PA levels at long-term [15, 40]. For example, the study by Ramadi et al. [35] showed that the exercise level increased similarly in the center-based and home-based groups during the program period. However, from post-CR to the 1-year follow-up, exercise capacity remained unchanged in the home-based CR participants, whereas the center-based CR group demonstrated a decline in exercise capacity. Based on this finding, a program that favors the transition between the cardiac rehabilitation center and home appears to be needed.

### Study Objectives

#### Primary research objective

We hypothesize that a progressively autonomous PA (PAPA) intervention for people with cardiovascular disease that promotes habit formation in an everyday-life context will result in better PA maintenance than a fully supervised PA (SPA) intervention. We do not expect any difference between groups immediately after the end of the intervention, but we assume that the difference will emerge over time, with the PAPA group showing a slower and smaller decline in PA behaviors after the end of the PA intervention than the SPA group.

#### Secondary research objectives

Exploratory analyses will assess the differences between the two groups in motivation for PA, automaticity of PA behavior and quality of life. We will also compare the evolution in the patients’ physical condition. Last, we will evaluate the cost-effectiveness of the two interventions. We hypothesize that the PAPA program promoting habit formation will result in better physical condition, motivation, automaticity of PA behavior and quality of life at the end of follow-up compared with the SPA program. We also hypothesize that the PAPA program will cost less than the SPA program.

## Method

The trial, registered as ISRCTN77313697, is a monocentric, two-arm, randomized, open-label study in cardiovascular patients to compare habit formation in a standard SPA-based CR program and a PAPA program.

### Sample size

The sample size was calculated using the simulation method of PASS 14 software (NCSS, Kaysville, USA) for power analysis with a mixed model. An alpha risk of 5 % and a bilateral hypothesis were taken into account for the power analysis. We estimated the expected scores of the participants in each arm of our study on the basis of the results of large-scale studies [34, 35, 38] that assessed PA behavior maintenance after CR with a questionnaire providing an average score of PA minutes per week. Given the linear effect of time, the change in PA behaviors after SPA suggested that the weekly time spent in PA behavior would be reduced by 5 min very month following the intervention. This result was used to infer the PA level of the group at each measurement point post-program. Several studies have shown that interventions designed to promote maintenance can lead to stabilized or even slightly increased PA behaviors over time (e.g., [2, 29, 34, 38]). We therefore predicted no change in the amount of PA behavior for the PAPA group after the intervention. The power analysis used a similar average score immediately after the intervention of 200 min/week for both groups, a general between-subject variability of 90 min/week, and an autocorrelation of the repeated measures of .55. (Fournier M, D'Arripe-Longueville F, Radel R. (unpublished manuscript). Testing the effect of text messaging cues to promote physical activity habits: A worksite-based exploratory intervention). The results after 1000 simulations indicated that 50 evaluable participants (25 participants in each arm) would be required to find the time-by-condition interaction effect with an 80 % power level. Assuming about 10 % of patients mistakenly included, lost to follow-up or withdrawing from the study, we randomized 56 participants (28 participants in each of the two arms).

### Participants

#### Recruitment strategy

The study is being conducted in the Alpes-Maritimes region of southern France; it began in 2014 and will run until 2016. CVD patients are identified from the electronic records of a public health insurance company for independent workers. The insurance company initially contacts the patients (*N* = 2248) by email, providing a detailed description of the project including the research objectives, subject requirements, timetable and the testing and training facilities. Potential participants are invited to contact the insurance company if they are interested in the experiment. Phone screening was conducted with 91 volunteers in October 2014. Then, they will have a free medical checkup with the study cardiologist, including a maximal effort test and an electrocardiogram.

#### Inclusion criteria

We restricted the study to adults over 18 years old, registered in the health insurance database with chronic disease (cardiovascular disease or cardiac deficiency) and considered as sedentary according to a brief physical activity assessment [27]. Further eligibility criteria include no contraindication to PA as certified by a cardiologist and ability to attend a 60-min session twice a week at a fitness center. The final sample will comprise 56 patients recruited on a voluntary basis.

### Consent

Consent to enter the study will be signed by each participant after they receive a full explanation in person, with the opportunity to ask questions. All participants have the right to decline participation or withdraw from the study at any time without giving a reason.

### Randomization

Randomization will be performed according to a 1:1 scheme, using the CS randomization Clinsight software module (Ennov, San Francisco, CA, USA). The method of randomization by minimization will be used to avoid imbalances between the two groups for all known and unknown patient-related factors that may influence the study outcome. Patient age (<65 years, ≥ 65 years), type of disease (coronary disease, cardiac insufficiency) and gender (Male, Female) will be stratified.

### Blinding

For practical reasons concerning the behavioral nature of the manipulation, strict participant blinding cannot be ensured. Several strategies will be implemented to prevent contamination and limit the potential biases associated with the lack of blinding [9].

In a randomized controlled trial in which the intervention is behavioral, hiding the content of the other study arm for participant blinding is difficult because true placebo conditions are unfeasible. To address this limitation, we adopted the double-consent procedure initially introduced by Zelen [47] and further adapted for behavioral intervention trials in patients with chronic disease [11]. In accordance with this procedure, eligible participants will first sign a general consent form and, after randomization, they will receive a second consent form detailing all the terms associated with their type of treatment without receiving any information about the study requirements concerning the other arm. It should be noted that a systematic review of the studies that have employed this procedure [1] indicated that this limited participant disappointment and increased the validity of the studies.

The investigators and PA coaches will be blinded to group assignment. The trial adheres to established procedures to maintain separation between the staff in charge of outcome measurements and the staff that will deliver the intervention. Staff members who obtain outcome measurements are not informed of the group assignment. In addition, those responsible for data analysis will be blinded to group status.

### Ethical approval

Ethical approval for the trial was received from the National Ethics Committee for Human Research (ref: 14 073) and the National Drug Agency (141299B-21). The regional governmental health agency also approved the various locations for the intervention (DOS-01115-0577-D).

### Intervention

The PAPA group will participate in two supervised sessions per week for the first 2.5 months and only one supervised session per week for the last 2.5 months. For the first 2.5 months, these patients will be advised to carry out at least one more session during the week on their own to meet the American College of Sports Medicine (ACSM) guidelines for cardiac patients [28]. However, they will be encouraged to carry out two autonomous sessions per week for the last 2.5 months. The supervised sessions will always be held at the same time of day and on the same day of the week with the same instructor. These sessions will include one session of Nordic walking (45 min to 1 h) at low to moderate intensity and one session of circuit training (1 h) at moderate to high intensity. The intensity levels will be defined in accordance with ACSM guidelines [28]. The session contents will be individualized according to each patient’s heart rate using a heart rate monitor (Polar FT1 heart-rate monitor watch). After controlling the resting heart rate and maximal heart rate, training windows will be calculated for each participant and used during the PA sessions. This will allow them to work at different intensities. The instructor will be trained to deliver an autonomy-supportive coaching style [41] as autonomous motivation has been shown to strengthen habit formation [19]. The tools we will use and the strategies we will implement are all aimed at supporting exercise instigation in terms of planning and preparatory behaviors (Barz et al. [4]). The goal is to develop autonomy, self-confidence and knowledge so that the participants can exercise by themselves in their homes. An individualized exercise prescription based on heart rate and current exercise capacity will be a core component of the intervention. Autonomous practice will be promoted in this group with support to aid habit formation. First, they will receive a pamphlet on PA (i.e., tips on safety rules, health benefits, nutrition, stretching, how to use a heart rate monitor, how to breathe) and a PA program containing a list of Nordic walking and cardio-training activities that can be done in their home environment. They will also receive the calendar used by Gardner et al. [20] that can be used as a tool for forming new habits. The calendar is generic and patients will be invited to use it in planning when and where they will do their PA sessions; they will thus be expected to write session plans down on the calendar. They will be advised to put the calendar in a place where they will see it every day (e.g., on the fridge and/or a bathroom mirror) so that they will have frequent reminders of their PA session. They will then be called every 2 weeks for an approximately15-min conversation with a researcher trained in habit formation theory. This researcher will help the patients to choose an appropriate moment and an appropriate context for the autonomous PA sessions and remind them to stick with their plans.

**Fig. 1:**
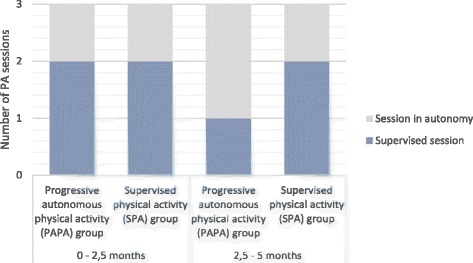
Illustration of the intervention showing the number of supervised sessions and autonomous sessions in the progressively autonomous (PAPA) group and the supervised (SPA) group

The SPA group will have two supervised sessions over 5 months. They will also be advised to carry out at least one more session on their own during the week to meet with the American College of Sports Medicine (ACSM) guidelines for cardiac patients [28] (Fig. 1).

### Outcome measures

#### Demographics

Demographic (gender, birthdate, zip code) and medical (cardiovascular disease or cardiac deficiency) data will be collected at baseline.

#### Behavioral measures

PA behavior will be assessed using a self-report questionnaire extensively used in the literature, the International Physical Activity Questionnaire (IPAQ) [14] at time (T) 0, T5, T7, T9 and T12 months. The questionnaire responses will be computed as energy requirements defined as METs (multiples of the resting metabolic rate). Based on previous research in other chronic diseases (e.g., [31, 32]), a subsample using a GT3X+ accelerometer will comprise 30 % of the participants selected randomly from among volunteers. According to this meta-analysis, an acceptable level of agreement between IPAQ and GT3X+ indicated a small to moderate association (r = .29). They will wear the GT3X for one week (score in METs) at T0, T5 and T12 in order to check whether the self-reported PA measure correlates with the objective PA measure in the context of our study. The PA adherence will be assessed by reporting on the participation in the two supervised sessions per week and the autonomous session.

#### Physical measures

The New York Heart Association (NYHA) test will be measured at T0 and T12 by the cardiologist to assess cardiovascular health [6]. Patients with heart failure will answer four questions in relation to how they feel during rest or physical activity. They will then be placed in one of four categories based on how limited they are during physical activity; the limitations/symptoms regard normal breathing and the varying degrees in shortness of breath and/or angina pain. General physical condition will be measured at T0, T5 and T12 with the 6-min walking test [30], a handgrip strength test [37] and the sit to stand test [7].

#### Psychological measures

All psychological measures will be made at T0, T5 and T12 months. The strength of PA habits will be measured using the Self-Report Behavioral Automaticity Index (SRBAI) [20]. We used a French adaptation of these items that was validated by Boiché [8]. The SRBAI is a four-item questionnaire (“I do automatically”; “I do without thinking”; “I do without having to consciously remember”; and “I start doing before I realize”) that is typically used by calculating the average automaticity score. Self-determined motivation will be assessed using the sport motivation scale [10] to rate each participant’s motivation regarding PA behavior. We will score the scale’s seven types of motivation according to self-determination theory: (a) amotivation; extrinsic motivation with (b) external regulation, (c) introjected regulation, (d) identified regulation, (e) integrated regulation and (f) intrinsic regulation (Deci & Ryan [17]). Last, we will assess the eventual changes in quality of life using the SF-36 questionnaire [44], which measures life quality in the domains of physical functioning, role-physical, bodily pain, general health, vitality, social functioning, role-emotional and mental health.

#### Economic measures

The quality-adjusted life-year (QALY) will be calculated at T0, T5 and T12 in order to assess the predicted increase in life expectancy. This widely used indicator is a measure based on the preferences expressed by the patients themselves with respect to different health states. This assessment allows for the adjustment in life years gained by the quality of life rated on a five-dimension scale, the “EQ5D” [13]. Participants in the experiment will be compared with a control group composed of the 78 individuals who initially volunteered to participate in the study but then realized they could not do so for several reasons: they were unavailable for the entire study, had changed their minds or had problems commuting to the PA sessions. To improve the reliability of the QALY, we will also include data on the number and duration of hospitalizations during the protocol and health care consumption; this information will be provided by the reimbursement databases for the year preceding inclusion and at 5 months of intervention and 6 months of follow-up.

We will also assess program cost by financially assessing the time spent by the various stakeholders, the travel cost for patients, and the cost of materials and fitness center rental space.

#### Statistics and data analysis

All statistical analyses will be performed with a 5 % alpha risk or 95 % confidence interval using SAS 9.3 (SAS Institute, Cary, NC, USA) or SPSS (IBM Statistics, Armonk, NY, USA). No interim analysis will be performed. The normality of quantitative data will be evaluated using a graphical method (frequency histogram and quantile-quantile plot) and a Shapiro-Wilk test [21]. To test the primary hypothesis on PA behavior maintenance, a mixed model procedure will be conducted to take into account the non-independence of the repeated IPAQ measures grouped within participants and the non-independence of the participants grouped in different centers. It should be noted that this type of analysis is highly recommended for repeated measures analysis [39, 48]. The repeated measures will be considered as a longitudinal fixed factor (measurements at T5, T7, T9 and T12). The condition (PAPA vs. SPA) representing the criterion of the analysis will be considered as a fixed effect in the model. The interaction between these two factors will be examined to test whether the change in PA behaviors after the end of the intervention differs depending on the arm of the trial. To control for individual differences in PA levels, the initial level of PA (IPAQ at T0) will be included as a fixed covariate. The intercept will be defined as a random factor that can vary for each participant and for each study center.

For the secondary objectives, we will first check for incomplete or missing data. Missing data at random will be imputed using the multiple imputations method. Sensitivity analyses will be used to address the impact of imputation on conclusions. A general linear model (GLM) will test the hypotheses, including the time of measurement (within-subject factor), the experimental condition (between-subject factor) and the interaction between these two factors.

## Discussion

Cardiovascular patients are generally willing to take part in CR, but unfortunately they do not systematically have the opportunity to do so (80 % of our sample). The PAPA intervention is designed to complement secondary prevention programs or replace them for those who cannot easily participate. The intervention aim is to provide cardiovascular patients with an opportunity to progressively learn, adopt and maintain PA behaviors.

The data from this study will be used to assess the impact of the *As du Coeur* intervention just after the end of the intervention (T5). We do not expect significant differences between the PAPA group and the fully supervised group at this point. Indeed, the fully supervised group will have more supervised sessions that the autonomous group (two per week versus one per week in the second half of the intervention). Based on the linear trend in the PA measures made in the months following the end of the intervention, we expect to find a decline in PA (negative linear slope of the time effect) post-intervention only for the SPA group. We expect that patients of the PAPA group will either maintain the same level (null slope) or even increase their PA (positive slope) in the months following the end of the intervention. Despite our low sample size due to a limited recruitment capacity, it is important to recall that this hypothesis can still be adequately tested with reasonable power using a mixed model approach, as simulation studies of the power in mixed model have shown that it typically increases with repeated measurements [23]. However, we will carry out exploratory analyses to test whether the PAPA intervention also leads to greater automaticity and motivation for PA, higher quality of life and better physical condition compared with the SPA intervention. Another limitation of the study can be the used of the sport motivation scale to assess participant’s motivation in the context of PA as motivation for sport and physical activity can differ.

As this study will show whether the PAPA intervention promotes physical activity maintenance in people with CVD, it has potentially significant implications for the management of cardiovascular patients. If successful, it will increase PA behaviors and hence reduce medical costs and enhance the quality of life of people with CVD over the long term. In addition to improving health outcomes in the short and long runs, this intervention has the potential to reduce the public health costs of cardiac rehabilitation. It therefore may prove to be a feasible addition to existing health services and could be delivered to people with CVD who have already attended CR programs as a “transition” program.

## Abbreviations

CR, cardiac rehabilitation; CVD, cardiovascular disease; PA, physical activity; PAPA, progressive autonomy; SPG, supervised physical activity
